# Supplementary material for: Cholesterol-rich naked mole-rat brain lipid membranes are susceptible to amyloid beta-induced damage in vitro
Source: Aging (Albany NY). 2020 Nov 4;12(21):22266–90. doi: 10.18632/aging.202138 (PMC7695401; doi:10.18632/aging.202138)
Supplement: Supplementary Methods [file aging-12-202138-s001..pdf]

SUPPLEMENTARY METODS

Production of naked mole-rat amyloid beta

The single point mutation (His13Arg) was introduced to the pET-Sac vector containing a gene encoding for wild type, human Aβ(M1–42) using a QuikChange Site Directed Mutagenesis kit (Agilent UK Ltd., Cheshire, UK) and following the manufacturer's protocol. The primers were: Forward-5'-GGTTACGAAGTTCGC CACCAGAAGCTGG-3' and Reverse - 5'-CCAG CTTCTGGTGGCGAACTTCGTAACC-3'. Plasmid sequences were confirmed by DNA sequencing (Dept. of Biochemistry, University of Cambridge, UK). The recombinant Naked Mole-Rat Aβ(M1-42) peptide (MDAEFRHDSGY EVRHQKLVFFAEDVGSNKGA IIGLMVGGVV IA), here called NMR amyloid beta, was expressed in the E. coli BL21 Gold (DE3) strain (Stratagene, CA, U.S.A.) and purified as described previously with slight modifications[1]. Briefly, the purification procedure involved sonication of E. coli cells, dissolution of inclusion bodies in 8 M urea, and ion exchange in batch mode on diethylaminoethyl cellulose resin followed by lyophylisation. The lyophilised fractions were further purified using Superdex 75 HR 26/60 column (GE Healthcare, Buckinghamshire, U.K.) and eluates were analysed by SDS-PAGE using 4-12% Bis-Tris NuPAGE gels and MES buffer (Thermofisher, Paisley, UK) for the presence of the desired protein product. The fractions containing the recombinant protein were combined, frozen using liquid nitrogen, and lyophilised again. The mass of the NMR amyloid beta was confirmed by mass spectrometry (Department of Chemistry, University of Cambridge, UK) (calculated mass: 4664.3 Da; observed mass: 4663.4 ± 0.8 Da).

Thioflavin T (ThT) kinetic assay

A sample of lyophilised naked mole-rat amyloid beta was dissolved in 1 ml GdnHCl (6 M, pH 8) and incubated on ice (1.5-2 hr). SEC was performed on the NMR Aβ42 using a Superdex 75 10/300 GL column (GE Healthcare, Amersham, UK). The buffer used for elution was a pH 8 sodium phosphate buffer (25mM Na_2PO_4, 0.2mM EDTA, pH 8) (flow rate 0.7 ml/min). Solutions containing monomer were collected and monomer concentrations were determined by the UV absorbance of the solution ($\varepsilon_{280\ nm}$ =1490 $M^{-1}cm^{-1}$). The monomer obtained in this way was diluted with buffer to the desired concentration and supplemented with 20 µM Thioflavin T (ThT) from a 1 mM stock. All samples were prepared in low binding eppendorf tubes on ice using careful pipetting to avoid introduction of air bubbles. Each sample was then pipetted into multiple wells of a 96-well half-area, low-binding, clear bottom and PEG coated plate (Corning 3881), to give 80 µL per well. Kinetics assays were initiated by placing the 96-well plate at 37 ºC under quiescent conditions in a plate reader (Fluostar Omega, BMGLabtech, Offenburg, Germany). The ThT fluorescence was measured through the bottom of the plate with a 440 nm excitation filter and a 480 nm emission filter. The ThT fluorescence was followed for three repeats of each sample.

Transmission electron microscopy

5µl of NMR and human Aβ42 aggregate solutions were applied to carbon-coated copper grids, stained with 2% (w/v) uranyl acetate and washed twice with 5 µl MilliQ water. The grids were then imaged with a Thermo Scientific Talos F200X G2 200 kV FEG Scanning Transmission Electron Microscope (Department of Chemistry, University of Cambridge).

SUPPLEMENTARY REFERENCE

1. Hellstrand E, Boland B, Walsh DM, Linse S. Amyloid β-protein aggregation produces highly reproducible kinetic data and occurs by a two-phase process. ACS Chem Neurosci. 2010; 1:13–8. https://doi.org/10.1021/cn900015v PMID:22778803
