# Supplementary material for: Cholesterol-rich naked mole-rat brain lipid membranes are susceptible to amyloid beta-induced damage in vitro
Source: Aging (Albany NY). 2020 Nov 4;12(21):22266–90. doi: 10.18632/aging.202138 (PMC7695401; doi:10.18632/aging.202138)
Supplement: Supplementary Figures [file aging-12-202138-s002..pdf]

SUPPLEMENTARY FIGURES

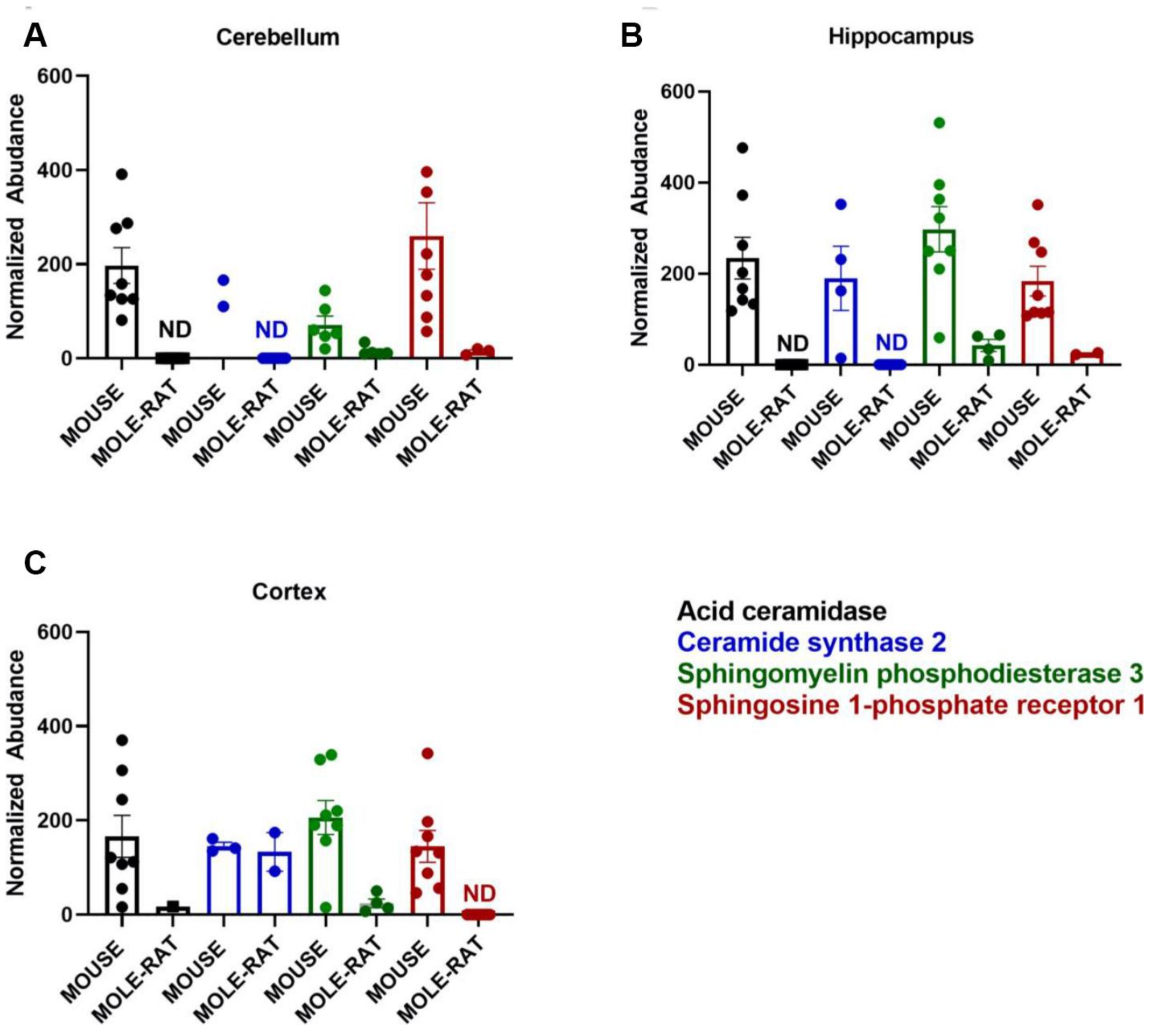

Supplementary Figure 1. Normalized abundance of proteins related to lipid metabolism and function in different brain regions in mouse and naked mole-rat. The normalized abundance of acid ceramidase, ceramide synthase 2, sphingomyelin phosphodiesterase 3 and sphingosine 1-phosphate receptor 1 are provided for cerebellum (**A**), hippocampus (**B**) and cortex (**C**) for mouse and naked mole-rat.

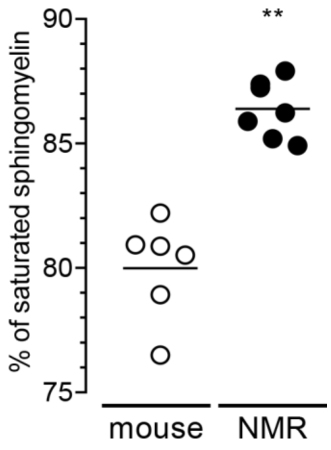

Supplementary Figure 2. Percentage of saturated sphingomyelin. Lipids were extracted from the brains of 7 naked mole-rats and 6 mice (2 independent experiments). % of saturated sphingomyelin in lipid extract from mouse (white circle) or naked mole-rat (black circle) brains. Data are represented as scatter dot plot with the mean. Statistical analysis was performed using the Mann-Whitney test. **p<0.01, significantly different from mouse group.

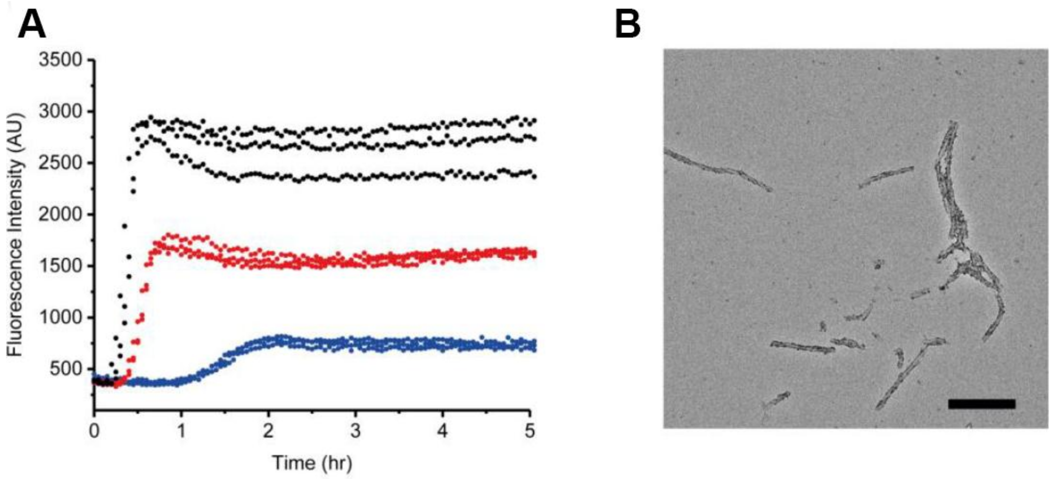

Supplementary Figure 3. (**A**) ThT kinetics traces for 1 µM (blue), 3 µM (red) and 5 µM (black) naked mole-rat amyloid beta shown in triplicate (pH 8, 37°C, quiescent conditions). Representative of two independent experiments. (**B**) TEM analysis of endpoint fibrils formed from kinetics assay (scale bar = 200 nm). Results indicative of a single batch of naked mole-rat amyloid beta.

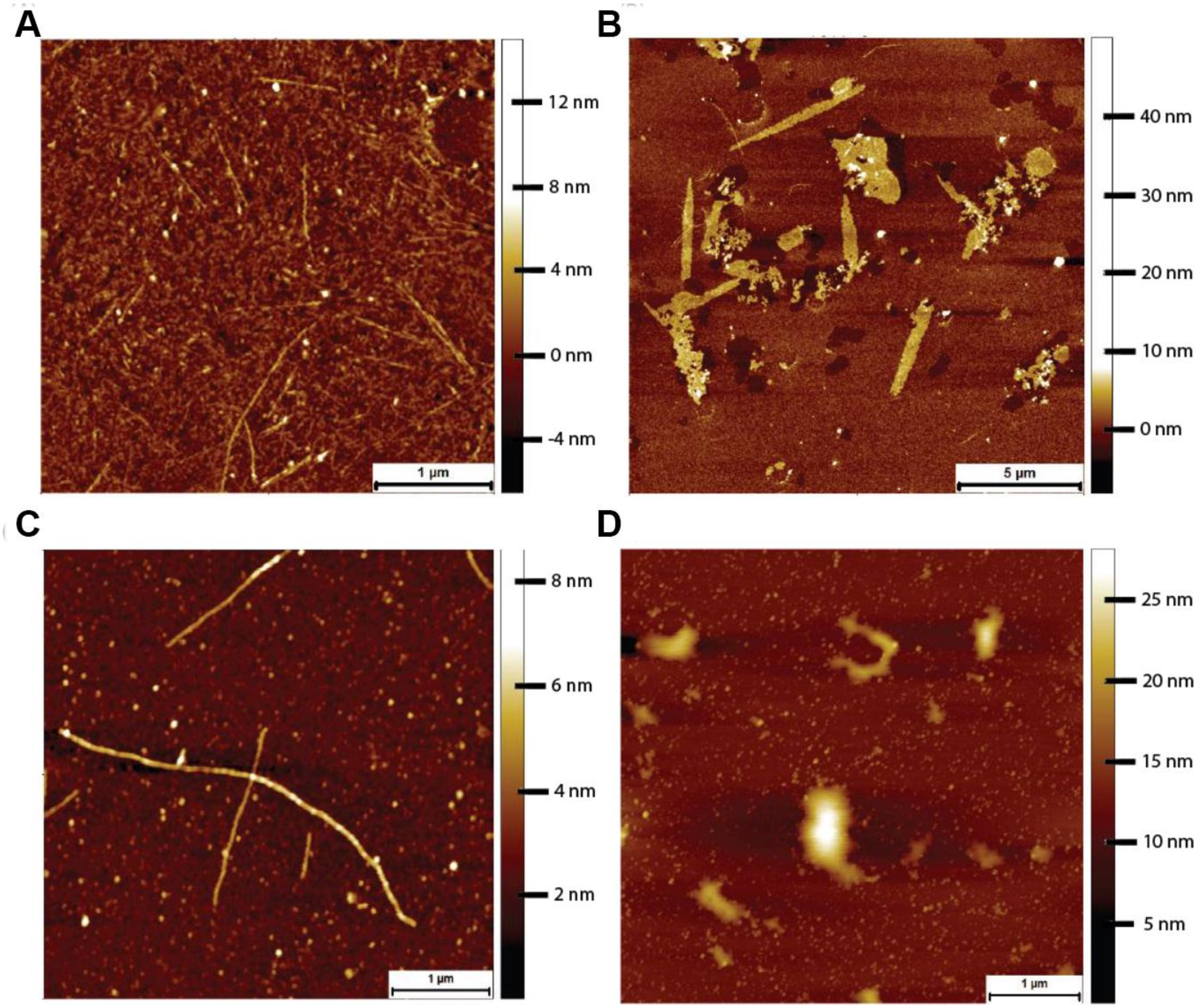

Supplementary Figure 4. Amyloid beta peptide adsorbed (air dried) onto mica at a concentration of 8 µM. (**A**) Synthetic human amyloid beta readily forms fibres. (**B**) Higher order structures of the human amyloid beta. These are well organised, and the constituent fibres are resolvable. (**C**) Recombinantly expressed naked mole-rat amyloid beta also forms fibres. (**D**) Less well-defined higher order structures are formed by naked mole-rat amyloid beta.

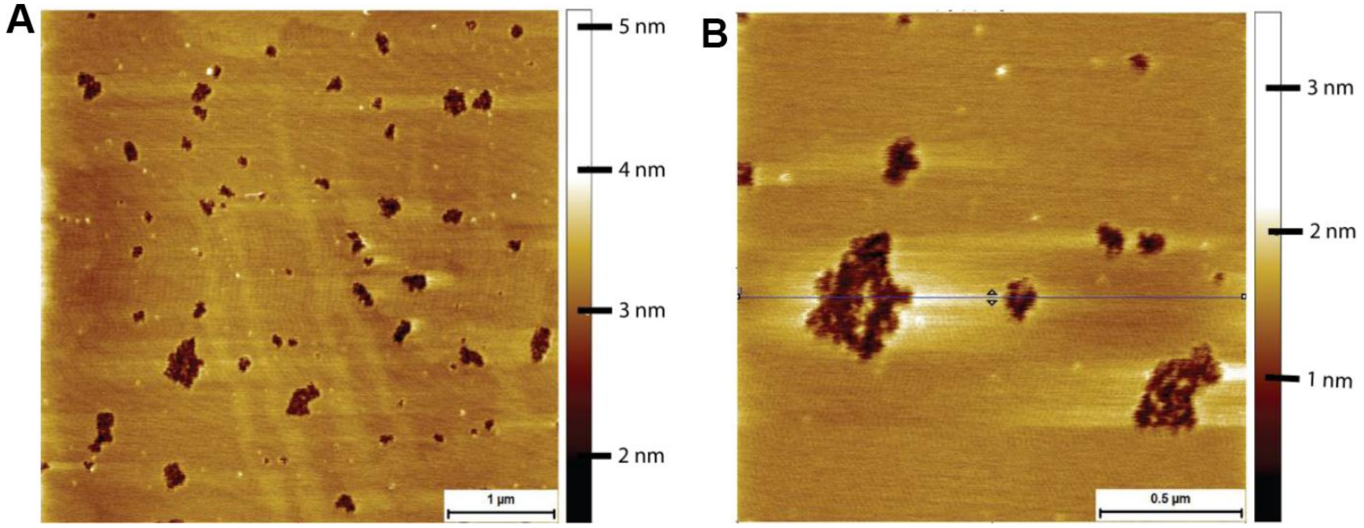

Supplementary Figure 5. AFM tapping mode images (in PBS) of mouse brain tissue derived supported lipid bilayers exposed to 8 µM of naked mole-rat amyloid beta for 2 hr. (**A**) Supported bilayer made from mouse brain derived lipids exhibiting naked mole-rat amyloid beta induced holes. (**B**) High resolution tapping mode image of naked mole-rat amyloid beta induced holes in mouse brain lipid derived bilayers. Holes do not penetrate bilayer.

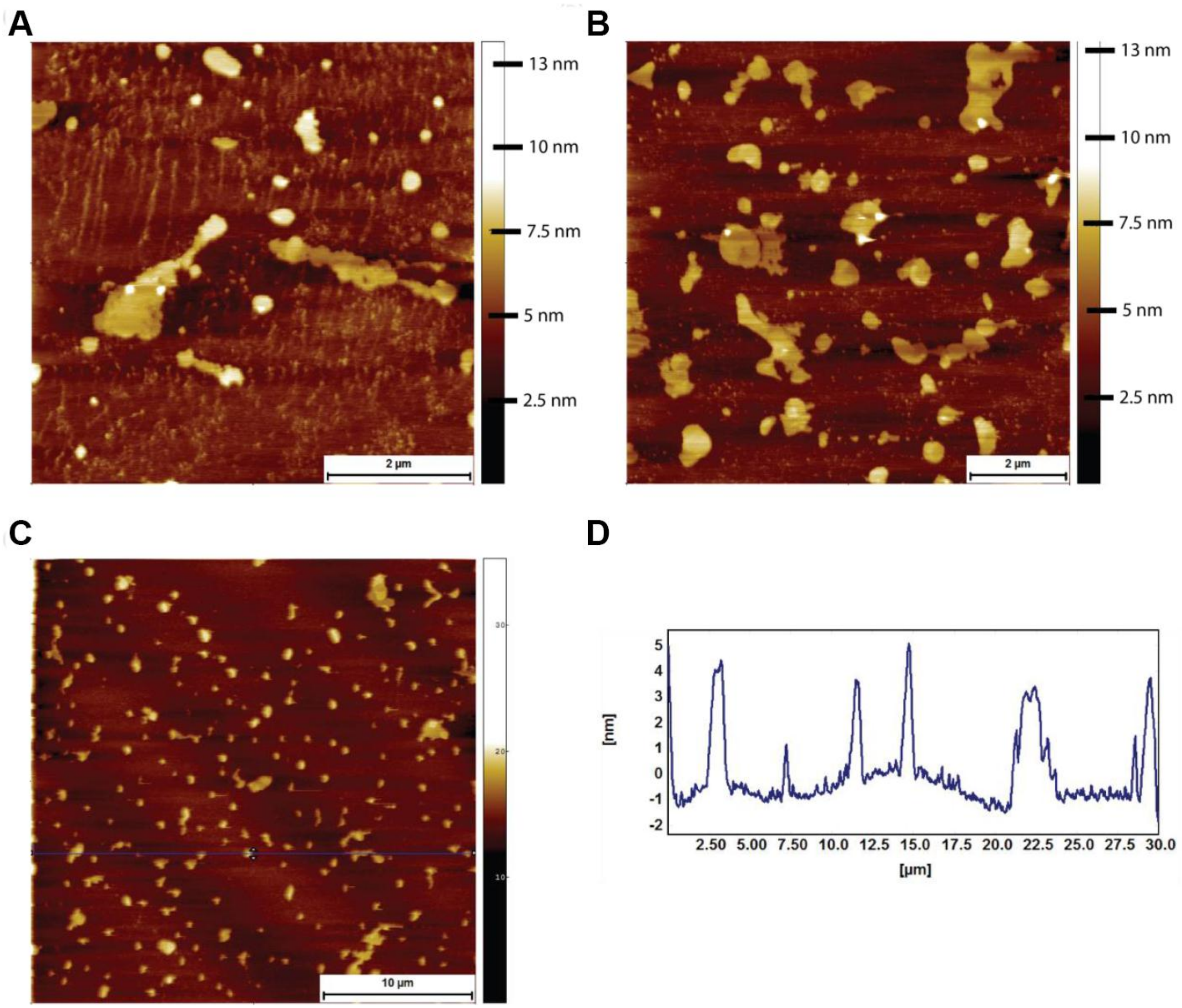

Supplementary Figure 6. AFM tapping mode images (in PBS) of naked mole-rat brain derived lipid bilayers exposed to 8 µM of naked mole-rat amyloid beta for 2 hr. (**A** and **B**) The bilayer is reduced to fragments of membrane, the height of fragments consistent with bilayer thickness. (**C**) Large scale topographic image showing the scale of the damage. (**D**) A height profile corresponding to the blue horizontal line in (**C**), confirming the height of fragments to be of bilayer thickness of 5-6 nm.
