# Supplementary material for: Cholesterol-rich naked mole-rat brain lipid membranes are susceptible to amyloid beta-induced damage in vitro
Source: Aging (Albany NY). 2020 Nov 4;12(21):22266–90. doi: 10.18632/aging.202138 (PMC7695401; doi:10.18632/aging.202138)
Supplement: Supplementary Tables [file aging-12-202138-s003..pdf]

SUPPLEMENTARY TABLES

Supplementary Table 1. percentage of phosphatidylcholine from mouse and naked mole rat brains.

	% of total phosphatidylcholine		
**Lipid number**	**Naked mole rat**	**Mouse**	**P value**
PC 28:0	0.87 ± 0.12	0.021 ± 0.003	0.0012
PC 30:0	8.80 ± 0.78	0.91 ± 0.10	0.0012
PC 30:1	2.70 ± 1.04	0.59 ± 0.26	0.2343
PC 32:0	22.04 ± 0.55	32.98 ± 0.58	0.0012
PC 32:1	5.00 ± 0.86	1.08 ± 0.24	0.0012
PC 32:2	0.14 ± 0.007	0.008 ± 0.0007	0.0012
PC 34:0	2.90 ± 0.09	3.41 ± 0.10	0.0082
PC 34:1	18.45 ± 0.61	17.67 ± 0.44	0.4452
PC 34:2	1.50 ± 0.10	0.66 ± 0.02	0.0012
PC 34:3	0.15 ± 0.01	0.02 ± 0.001	0.0012
PC 36:1	4.19 ± 0.09	7.68 ± 0.08	0.0012
PC 36:2	2.57 ± 0.19	2.98 ± 0.17	0.1014
PC 36:3	1.51 ± 0.06	1.20 ± 0.03	0.0012
PC 36:4	6.47 ± 0.12	5.69 ± 0.08	0.0012
PC 38:2	0.21 ± 0.02	0.50 ± 0.01	0.0012
PC 38:3	1.21 ± 0.02	1.07 ± 0.01	0.0012
PC 38:4	7.83 ± 0.35	6.93 ± 0.25	0.1375
PC 38:5	3.70 ± 0.10	2.43 ± 0.33	0.0012
PC 38:6	3.98 ± 0.05	5.68 ± 0.20	0.0012
PC 40:3	0.097 ± 0.002	0.08 ± 0.002	0.0140
PC 40:6	5.64 ± 0.07	8.35 ± 0.26	0.0012

Values are expressed as mean ± SEM; Statistical analysis was performed using Mann-Whitney test.

Supplementary Table 2. percentage of phosphatidylethanolamine from mouse and naked mole rat brains.

	% of total phosphatidylethanolamine		
**Lipid number**	**Naked mole rat**	**Mouse**	**P value**
PE 32:0	0.50 ± 0.01	0.39 ± 0.03	0.0140
PE 32:1	0.56 ± 0.07	0.18 ± 0.02	0.0012
PE 32:2	0.040 ± 0.004	0.007 ± 0.001	0.0012
PE 34:0	8.88 ± 1.12	8.39 ± 1.02	0.2343
PE 34:1	1.04 ± 0.15	0.57 ± 0.06	0.0221
PE 36:1	8.46 ± 0.64	14.50 ± 0.40	0.0012
PE 36:2	4.71 ± 0.76	7.32 ± 0.88	0.0350
PE 36:3	1.77 ± 0.11	1.31 ± 0.16	0.1014
PE 36:4	1.79 ± 0.09	1.42 ± 0.07	0.0140
PE 38:2	1.83 ± 0.17	1.90 ± 0.10	0.9452
PE 38:3	13.78 ± 2.55	8.26 ± 1.77	0.1014
PE 38:4	16.55 ± 0.22	11.46 ± 0.22	0.0012
PE 38:5	3.81 ± 0.43	5.25 ± 0.54	0.1014
PE 38:6	4.40 ± 0.24	4.77 ± 0.53	0.9452
PE 40:3	3.57 ± 0.65	1.96 ± 0.38	0.1014
PE 40:5	12.14 ± 1.35	13.0& ± 2.26	0.7308
PE 40:6	14.60 ± 1.66	17.85 ± 1.80	0.1014
PE 40:7	1.55 ± 0.30	1.42 ± 0.26	0.2343

Values are expressed as mean ± SEM; Statistical analysis was performed using Mann-Whitney test.

Supplementary Table 3. percentage of phosphatidylinositol from mouse and naked mole rat brains.

	% of total phosphatidylinositol		
**Lipid number**	**Naked mole rat**	**Mouse**	**P value**
PI 32:1	0.19 ± 0.02	0.147 ± 0.008	0.1014
PI 34:1	1.40 ± 0.19	2.08 ± 0.06	0.0350
PI 34:2	0.39 ± 0.07	0.38 ± 0.02	0.4452
PI 36:0	0.24 ± 0.02	0.66 ± 0.04	0.0012
PI 36:1	0.81 ± 0.09	2.10 ± 0.07	0.0012
PI 36:2	0.67 ± 0.08	0.90 ± 0.02	0.1807
PI 36:3	2.36 ± 0.11	2.58 ± 0.20	0.4452
PI 38:1	0.10 ± 0.01	0.285 ± 0.008	0.0012
PI 38:2	1.02 ± 0.04	1.04 ± 0.05	0.7308
PI 38:3	18.49 ± 1.08	17.34 ± 1.04	0.1375
PI 38:4	55.34 ± 0.38	52.74 ± 0.27	0.0012
PI 38:5	16.58 ± 0.87	14.51 ± 0.99	0.2343
PI 40:2	0.026 ± 0.001	0.032 ± 0.008	0.9452
PI 40:3	0.118 ± 0.003	0.159 ± 0.007	0.0012
PI 40:4	0.377 ± 0.009	0.51 ± 0.01	0.0012
PI 40:5	0.73 ± 0.05	1.39 ± 0.05	0.0012
PI 40:6	1.16 ± 0.15	3.13 ± 0.15	0.0012

Values are expressed as mean ± SEM; Statistical analysis was performed using Mann-Whitney test.

Supplementary Table 4. percentage of phosphatidylserine from mouse and naked mole rat brains.

	% of total phosphatidylserine		
**Lipid number**	**Naked mole rat**	**Mouse**	**P value**
PS 32:0	0.027 ± 0.001	0.011 ± 0.001	0.0023
PS 34:0	0.57 ± 0.02	0.48 ± 0.01	0.0047
PS 34:1	2.37 ± 0.12	1.58 ± 0.02	0.0012
PS 34:2	0.19 ± 0.01	0.091 ± 0.002	0.0012
PS 36:0	2.03 ± 0.16	3.27 ± 0.08	0.0012
PS 36:1	6.66 ± 0.36	12.22 ± 0.37	0.0012
PS 36:2	3.32 ± 0.14	4.88 ± 0.16	0.0012
PS 36:3	0.345 ± 0.007	0.188 ± 0.003	0.0012
PS 38:2	0.74 ± 0.03	0.86 ± 0.04	0.0350
PS 38:3	2.57 ± 0.10	1.84 ± 0.06	0.0023
PS 38:4	5.29 ± 0.07	3.71 ± 0.13	0.0012
PS 38:6	0.38 ± 0.04	0.61 ± 0.06	0.0221
PS 40:5	23.33 ± 0.43	18.02 ± 0.21	0.0012
PS 40:6	51.91 ± 0.43	52.01 ± 0.32	0.5338
PS 42:6	0.263 ± 0.009	0.23 ± 0.01	0.1807

Values are expressed as mean ± SEM; Statistical analysis was performed using Mann-Whitney test.

Supplementary Table 5. percentage of sphingomyelin from mouse and naked mole rat brains.

	% of total sphingomyelin		
Lipid number	Naked mole rat	Mouse	P value
SM 18:1/14:0	1.95 ± 0.07	0.113 ± 0.008	0.0012
SM 18:1/16:0	20.05 ± 0.95	4.16 ± 0.18	0.0012
SM 18:1/16:1	0.46 ± 0.05	0.131 ± 0.009	0.0012
SM 18:1/18:0	58.86 ± 0.68	68.62 ± 1.05	0.0012
SM 18:1/18:1	5.46 ± 0.54	5.79 ± 0.39	0.9452
SM 18:1/20:0	1.61 ± 0.14	2.53 ± 0.31	0.0734
SM 18:1/20:1	0.150 ± 0.009	0.35 ± 0.02	0.0012
SM 18:1/22:0	1.08 ± 0.18	1.54 ± 0.16	0.1014
SM 18:1/22:1	0.56 ± 0.05	0.83 ± 0.13	0.2949
SM 18:1/24:0	2.83 ± 0.33	3.03 ± 0.11	0.7308
SM 18:1/24:1	6.98 ± 0.77	12.90 ± 0.52	0.0012

Values are expressed as mean ± SEM; Statistical analysis was performed using Mann-Whitney test.

Supplementary Table 6. percentage of ceramide from mouse and naked mole rat brains.

	% of total ceramide		
Lipid number	Naked mole rat	Mouse	P value
Cer d18:1/16:0	12.39 ± 0.66	1.99 ± 0.06	0.0012
Cer d18:1/16:1	0.087 ± 0.003	0.018 ± 0.001	0.0012
Cer d18:1/18:0	77.92 ± 0.38	85.18 ± 1.70	0.0012
Cer d18:1/18:1	1.15 ± 0.07	0.91 ± 0.09	0.1375
Cer d18:1/20:0	0.76 ± 0.03	3.40 ± 0.08	0.0012
Cer d18:1/22:0	1.11 ± 0.06	1.35 ± 0.10	0.1014
Cer d18:1/24:0	3.06 ± 0.44	3.12 ± 0.71	>0.9999
Cer d18:1/24:1	3.45 ± 0.47	3.97 ± 0.99	0.8357
Cer d18:1/26:0	0.033 ± 0.008	0.023 ± 0.008	0.2343
Cer d18:1/26:1	0.038 ± 0.007	0.022 ± 0.006	0.1014

Values are expressed as mean ± SEM; Statistical analysis was performed using Mann-Whitney test.

Supplementary Table 7. Relative concentration of fatty acid from mouse and naked mole rat brains.

		Relative concentration per g of tissue		
Common name	Lipid number	Mouse	Naked mole rat	P value
Myristic acid	C14:0	0.84 ± 0.08	2.53 ± 0.19	0.0025
Palmitic acid	C16:0	0.73 ± 0.02	1.14 ± 0.05	0.0012
Stearic acid	C18:0	670.90 ± 69.27	526.00 ± 33.00	0.1375
Hypogeic acid	C16:1 n-9	3.03 ± 0.50	17.90 ± 2.14	0.0012
Palmitoleic acid	C16:1 n-7	8.62 ± 1.25	11.00 ± 1.13	0.2774
Elaidic acid	C18:1 n-9	540.60 ± 59.06	425.70 ± 21.36	0.1807
Vaccenic acid	C18:1 n-7	117.50 ± 13.06	110.20 ± 5.10	0.6282
Gondoic acid	C20:1 n-9	75.29 ± 11.01	35.49 ± 6.50	0.0140
Linoleic acid	C18:2 n-6	20.48 ± 2.86	22.66 ± 2.13	0.8357
α-Linolenic acid	C18:3 n-3	0.87 ± 0.14	0.87 ± 0.14	0.9452
Eicosadienoic acid	C20:2 n-6	8.77 ± 1.09	9.39 ± 1.03	0.7308
Dihomo-γ-linolenic acid	C20:3 n-6	19.39 ± 2.25	21.26 ± 1.79	0.5589
Arachidonic acid	C20:4 n-6	353.40 ± 42.06	360.10 ± 29.88	0.8357
Eicosapentaenoic acid	C20:5 n-3	1.11 ± 0.15	5.40 ± 0.89	0.0012
Docosatrienoic acid	C22:3 n-3	7.81 ± 1.17	24.20 ± 3.36	0.0023
Adrenic acid	C22:4 n-6	89.30 ± 11.63	93.05 ± 10.27	0.9999
Docosapentaenoic acid	C22:5 n-3	11.77 ± 2.09	38.43 ± 3.11	0.0012
Docosahexaenoic acid	C22:6 n-3	660.00 ± 88.74	382.30 ± 1.67	0.0221

Values are expressed as mean ± SEM; Statistical analysis was performed using Mann-Whitney test.
